# Serum MyomiRs as Biomarkers for Female Carriers of Duchenne/Becker Muscular Dystrophy

**DOI:** 10.3389/fneur.2020.563609

**Published:** 2020-09-18

**Authors:** Jiapeng Zhang, Qi Meng, Jingzi Zhong, Min Zhang, Xiao Qin, Xiaohua Ni, Jiawen Ma, Yangwen He, Dan Zeng, Dan Lan

**Affiliations:** Department of Pediatrics, The First Affiliated Hospital of Guangxi Medical University, Nanning, China

**Keywords:** duchenne/becker muscular dystrophy, female carriers, microRNAs, myomiRs, creatine kinase, biomarkers

## Abstract

**Background:** Duchenne/Becker muscular dystrophy (DMD/BMD) is an X-linked recessive lethal neuromuscular disease. MicroRNAs expressed in striated muscle, myomiRs, have been proposed as its potential biomarkers. Serum creatine kinase (CK) is commonly used as a biomarker in clinical practice, but it is not reliable. The aim of this study was to assess whether serum levels of myomiRs has diagnostic value for detection of female DMD/BMD carriers with normal or elevated CK.

**Methods:** Thirty four female carriers and 33 age-matched healthy female controls were enrolled. Peripheral blood samples were collected and serum miRNAs were extracted for measurement of miR-1, miR-133a, miR-133b, miR-206, miR-208a, miR-208b, and miR-499 by quantitative real-time polymerase chain reaction.

**Results:** MiR-1, miR-133a, miR-133b, miR-206, miR-208a, miR-208b, and miR-499 were upregulated in all female carriers in comparison to healthy controls. MiR-1 (Spearman's rho = +0.406, *p* = 0.017) was correlated with CK in the female carrier group. Receiver operating characteristic curve analysis of all seven myomiRs showed that the area under the curve (AUC) for miR-499, miR-133b, miR-1, miR-208b, and miR-133a exceeded 70.0%, and for miR-206 and miR-208a exceeded 60.0%. MiR-133b and miR-499 were significantly increased in all female carriers, even those with normal CK. AUC for the combination of all seven miRNAs was 87.2%. CK (OR 0.406, 95% CI 0.000–0.001, *p* < 0.0001) and miR-499 (OR 0.323, 95% CI 0.023–0.106, *p* = 0.003) were considered to be independent predictors for female carriers presence in the multivariable regression analysis model.

**Conclusions:** MiR-133b and miR-499 are potentially useful biomarkers for female carriers with DMD/BMD (including those with normal CK). The combination of all seven serum miRNAs and their respective combinations with CK have better diagnostic value for female carriers than either CK or any separate miRNA.

## Introduction

Duchenne/Becker muscular dystrophy (DMD/BMD) is an X-linked recessive lethal neuromuscular disease with an incidence of ~1 in 3,500–5,000 live male newborns and is characterized by progressive skeletal muscle weakness (1). It is caused by mutations in the dystrophin gene, and two thirds of cases are inherited from their mother. To date, as the disease has no effective cure, implementing strategic family planning based on available medical evidence might be a good approach to reduce disease incidence. Thus, it is of great importance to establish a correlation between the biological markers in mother and the incidence rate.

It is estimated that ~2.5–40.0% of female carriers will have manifestations of muscular dystrophy (MD) such as muscle weakness or heart disease later on in their life, but these people rarely show symptoms during childhood (2–4). In addition, multiple studies have shown that ~45–76% of female carriers have increased serum creatine kinase (CK) levels, while others are normal (5–7). For those asymptomatic carriers with normal CK levels, their affected male relatives are usually the first indication of their so-called “gene carrier” status (8). Multiplex ligation-dependent probe amplification (MLPA) and next-generation sequencing (NGS) can be used to diagnose the condition at the genetic level, but they have the disadvantages of being expensive and time consuming. Muscle biopsy is seldom performed as it is an invasive test, especially for asymptomatic female carriers. Cardiac magnetic resonance imaging (CMR) and muscle magnetic resonance imaging (MRI) are still at the exploratory research stage, and no clear conclusions can be applied to the detection of carriers. Currently, the most commonly used circulating biomarker is serum CK. However, approximately one-third of carriers have normal CK levels, and its level can fluctuate with physical activity, muscle injury, cramps, toxic substances, and age (9, 10). Therefore, it is highly desirable to explore more effective potential biomarkers for detecting female carriers of DMD/BMD.

MicroRNAs (miRNAs) are small non-coding RNA molecules that exist both in cells and the circulation, and play an important role in regulating gene expression at the post-transcriptional level (11–13). The levels of miRNAs in serum are stable, reproducible, and consistent among individuals of the same species (13, 14). MiRNAs which are exclusively or preferentially expressed in striated muscle are called myomiRs (15). The group mostly includes miR-1, miR-133a, miR-133b, miR-206, miR-208a, miR-208b, miR-486, and miR-499 (16–21). Li et al. (22) found that the serum levels of a group of miRNAs including miR-1, miR-133, miR-206, miR-208a, miR-208b, and miR-499 have important diagnostic and prognostic values for DMD patients. Anaya-Segura et al. (4) further researched and concluded that up-regulated serum miR-206 could be used to detect DMD female carriers. Mousa et al. (23) also confirmed that plasma miR-499 was significantly up-regulated and had the ability to identify female carriers. As most of the existing literature only revealed the role of myomiRs in DMD patients and only few of them detected part of myomiRs' role in DMD carriers, we aimed to study the ability of different myomiRs in diagnosing female DMD carriers in a larger sample size in order to prevent the birth of DMD babies to some extent. Hence, we assessed the expression patterns of the seven serum myomiRs (miR-1, miR-133a, miR-133b, miR-206, miR-208a, miR-208b, and miR-499) in DMD/BMD patients, carriers and normal controls, and found that DMD patients had the highest expression levels, followed by female carriers and the lowest expression was detected in normal controls. Although these data have not been formally published yet, they lay the foundation for our present study of the ability of circulating myomiRs to act as biomarkers in female carriers of DMD/BMD.

In the present study, we attempted to explore and evaluate the diagnostic value of miR-1, miR-133a, miR-133b, miR-206, miR-208a, miR-208b, and miR-499 for the detection of female DMD/BMD carriers with normal or elevated CK levels.

## Materials and Methods

### Study Population

Thirty-four females were genetically confirmed to be DMD or BMD carriers and their gene mutation results are listed in the Supplementary File. Collectively these subjects were referred to as “MD-carriers,” and 33 of which were DMD carriers and the other one was a BMD carrier (Table 1). The assessment of muscle strength and cardiac performance was mainly carried out by carefully asking the carriers for symptoms such as fatigue, muscle pain, exercise intolerance, chest tightness after exercise, dyspnea, and so on. Then the carriers' proximal upper, proximal lower, and distal lower extremity was evaluated according to the Medical Research Council (MRC) Scale (24). The control group consisted of 33 age-matched healthy females with normal CK levels, whose genetics showed them to be negative for MD and have no related symptom. The study protocol complies with the Declaration of Helsinki and was approved by the Ethics Committee of the First Affiliated Hospital of Guangxi Medical University, Nanning, PR China. Informed consent was obtained from all participants.

**Table 1:** Characteristics of the study population.

	**MD-carriers**	**Controls**	***p*-value**
Female, *n*	34	33	–
DMD/BMD, *n* (%)	33 (97.00)/1 (3.00)	–	–
Age, years	31.18 ± 8.88	26.76 ± 4.72	**0.014**
CK, U/L [median (IQR)]	311.00 (147.50–598.25)	72.00 (62.00–86.00)	**<0.0001**
CK < 178U/L, *n* (%)	10 (30.30)	33 (100.00)	**0.000**

*Both data of DMD/BMD and CK <178U/L were expressed in frequency (n) and percentage (%). The age of the study population conformed to a normal distribution and was expressed as mean ± standard deviation (SD), and the independent-sample t test was used for analysis. CK does not conform to the normal distribution and was expressed in median and interquartile range (IQR), and the non–parametric Mann–Whitney U-test was used for analysis. P-value in bold was statistically significant (p < 0.05)*.

### Blood Sampling in Carriers and Controls

Sera from blood samples in collection tubes were extracted on the day of collection and centrifuged at 3,000 rpm for 10 min. The sera were stored in clean enzyme-free cryotubes (Eppendorf, Germany) at −20°C until use. In addition, CK levels were determined using a spectrophotometric assay.

### MiRNA Extraction and Quantification

MicroRNAs were extracted from 200 μL of the participants' serum samples using the miRcute Serum/Plasma miRNA Isolation Kit (cat. no. DP503, Tiangen Biotech, Beijing, China) following the manufacturer's instructions, and were stored at −80°C. Using microRNAs as templates, cDNA was reverse transcribed using miRcute Plus miRNA First-Strand cDNA Kit (cat. no. KR211, Tiangen Biotech). The final reverse transcription reaction volume of 20 μL contained 2μL miRNA RT enzyme mix [*E.coli* Poly(A) polymerase, RTase and RNasin], 10 μL of 2 × miRNA RT reaction buffer and 8 μL of total RNA. The reaction was performed following the manufacturer's protocol. The miRNA expression levels were quantified by reverse transcription polymerase chain reaction (RT-PCR) using miRcute Plus miRNA PCR Kit (SYBR Green) (cat. no. FP411, Tiangen Biotech) on the Applied Biosystems 7500 Fast Real-Time PCR System (Applied Biosystems, Foster, California, United States) following the manufacturer's protocol. Relative levels of miRNA expression were calculated by normalization to expression levels of an external control for miRNAs (cat. no. CR100-01, Tiangen Biotech). The 2^−ΔΔCt^ method was used to determine relative miRNA levels, where Ct is the threshold cycle. Differences in the expression levels of the seven miRNAs in the MD-carriers were compared with the control subjects and were expressed as -fold changes.

### Statistical Analysis

Processed variables that were normally distribution were expressed as mean ± standard deviation (SD), and variables that were skewed were expressed as median and interquartile range (IQR). Categorical variables were expressed as frequency with a percentage. The independent sample *t*-test was used for comparison of normally distributed variables, and the non-parametric Mann–Whitney *U*-test was used for comparison of non-normally distributed variables. Non-parametric Kruskal–Wallis *H*-test was used to perform multiple comparisons of non-normally distributed variables. Correlation analysis used the parametric Pearson correlation coefficient and the non-parametric Spearman correlation coefficient. Analysis of the receiver operating characteristic curves (ROC) were used to evaluate the specificity and sensitivity of each miRNA as a serum biomarker for detecting MD-carriers. The ROC curve is known as a plot of a test's false-positive rate (FPR), or 1-specificity (plotted on the horizontal axis), vs. its sensitivity (plotted on the vertical axis) (25). Specificity (the true negative rate) tells us how good the test is at defining people without the disease correctly. Sensitivity (the true positive rate) tells us how good the test is at finding people with the condition investigated. (26). Youden's index (sensitivity+specificity-1) (25), corresponding to the optimal sensitivity and specificity, is used to identify the optimal cut-off point. The area under the curve (AUC) represents a generalized measure of accuracy, and its value ranges from 0.5 (chance) to 1.0 (perfect discrimination or accuracy) (25). When combining multiple myomiRs and CK for ROC analysis, binary logistic regression was used to calculate the predicted probability values of multiple indicators, and the predicted probability values were used as the test variable for ROC analysis. In order to initially screen out the presence of independent predictors for MD-carriers, we first performed a binary logistic regression analysis. Subsequently, we introduced parameters with significant *p*-values into the multivariable regression analysis. Statistical analysis was performed using SPSS software (version 25.0, SPSS, Chicago, Illinois, United States) and GraphPad (version 8.0.2, GraphPad, San Diego, California, United States) for Windows. All *p*-values were two-sided, and *p* < 0.05 was considered statistically significant.

## Results

### Characteristics of the Study Population

**Figure 1:**
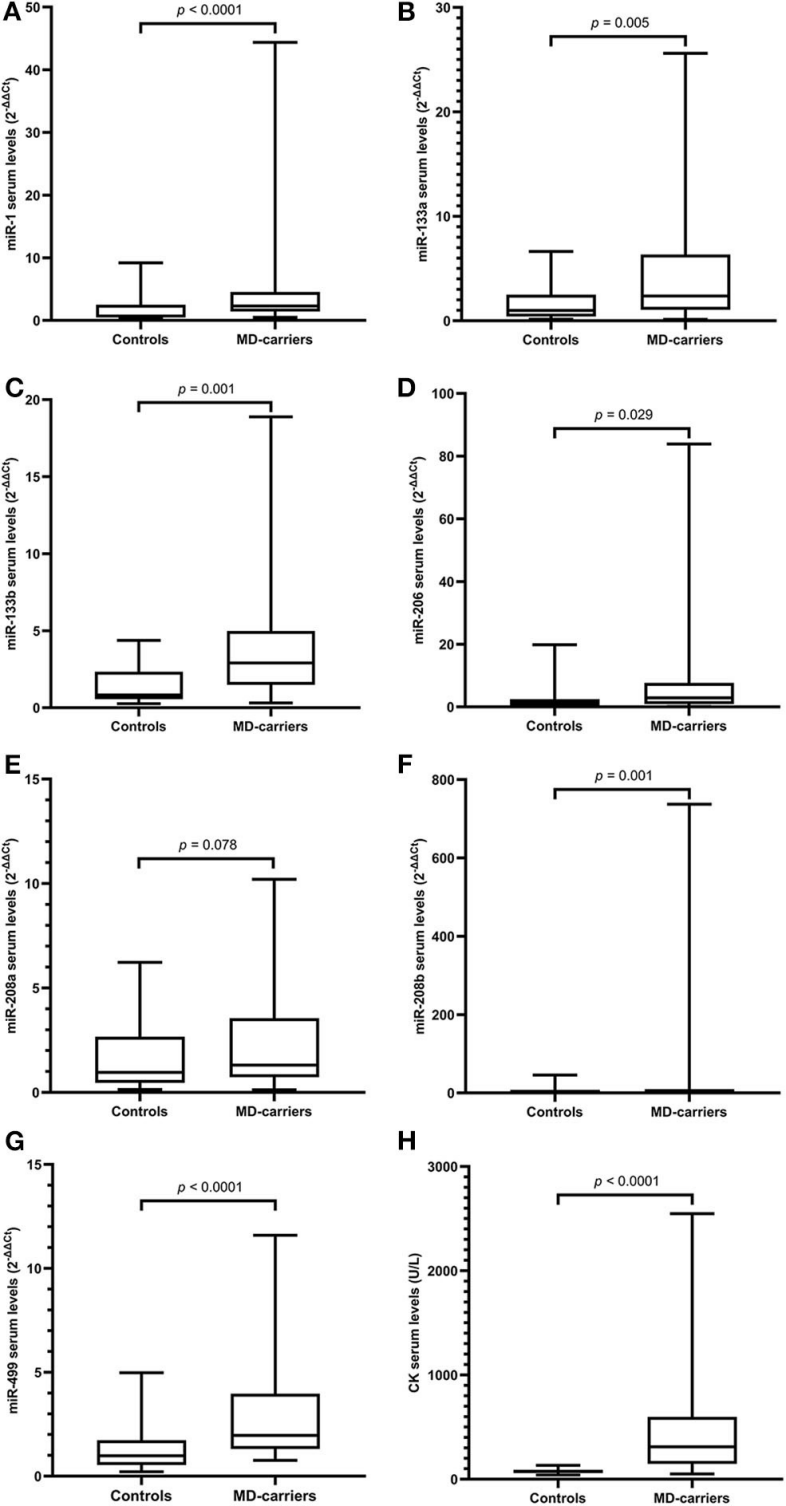
Levels of microRNAs and CK in serum of MD-carriers (*n* = 34) compared to controls (*n* = 33). **(A–H)**; miR-1, miR-133a, miR-133b, miR-206, miR-208a, miR-208b, mir-499, and CK, respectively. As data of both the carrier and control group were non-normally distributed variables, the non-parametric Mann–Whitney *U*-test was used for comparison between the two groups. *P* < 0.05 was considered statistically significant.

The general characteristics for the MD-carriers (*n* = 34) and female control group (*n* = 33) are shown in Table 1. All MD-carriers and controls had no related MD manifestations or heart involvement such as fatigue, myalgia or exercise intolerance, and their muscle strengths were recorded as 5/5 according to the MRC scale. The MD-carriers are comprised of 33 (97.00%) carriers with DMD and 1 (3.00%) with BMD with a median age of 31.18 ± 8.88 years (*p* = 0.014). Moreover, the MD-carriers had significantly higher average CK levels compared to controls (4.32-fold, *p* < 0.0001, Figure 1). In the MD-carriers, 10 (30.30%) of them exhibited a CK level below the upper limit of the normal range (<178U/L).

### Serum Levels of MyomiRs in MD-Carriers and Controls

Serum miRNA results in MD-carriers and controls are shown in Table 2. A varying degree of up-regulation in MD-carriers compared to controls was found in all serum miRNAs included in the analysis (Figure 1): miR-1 (3.23-fold increase, *p* < 0.0001), miR-133a (2.40-fold, *p* = 0.005), miR-133b (3.50-fold, *p* < 0.0001), miR-206 (2.46-fold, *p* = 0.029), miR-208a (1.36-fold, *p* = 0.078), miR-208b (3.41-fold, *p* = 0.001), and miR-499 (2.00-fold, *p* < 0.0001).

**Table 2:** Serum miRNA levels in MD-carriers compared to control subjects.

**miRNA serum levels (2^**−**^^**ΔΔ**^^**Ct**^) [median (IQR)]**	**MD-carriers (*n* = 34)**	**Controls (*n* = 33)**	***p*-value**
miR-1	2.30 (1.40–4.56)	0.71 (0.46–2.50)	**<0.0001**
miR-133a	2.38 (1.07–6.36)	0.99 (0.41–2.51)	**0.005**
miR-133b	2.91 (1.49–4.99)	0.83 (0.56–2.34)	**<** **0.0001**
miR-206	2.91 (0.88–7.65)	1.18 (0.38–2.48)	**0.029**
miR-208a	1.31 (0.73–3.56)	0.96 (0.46–2.67)	0.078
miR-208b	4.30 (1.78–8.77)	1.26 (0.19–3.34)	**0.001**
miR-499	1.96 (1.31–3.97)	0.98 (0.54–1.73)	**<** **0.0001**

*The serum levels of miRNAs in the two groups did not conform to the normal distribution and their data were described by the median and IQR. The non-parametric Mann–Whitney U-test was used for comparison between the two groups. P-value in bold was statistically significant (p < 0.05)*.

In addition, the carriers were divided into four groups according to the types of gene mutation sites, and the expression levels of these seven miRNAs were compared between the four groups. The results showed no significant difference (*p* > 0.05, Table 3).

**Table 3:** Comparison of serum miRNA levels in MD-carriers with different gene mutation.

**miRNA serum levels (2^**−**^^**ΔΔCt**^) [median (IQR)]**	**Deletion mutation (*n* = 20)**	**Duplication mutation (*n* = 5)**	**Point mutation (*n* = 5)**	**Splice mutation (*n* = 4)**	***p*-value**
miR-1	2.21 (1.29–4.89)	2.30 (1.58–4.64)	3.86 (3.35–11.19)	1.18 (0.78–1.46)	0.3203
miR-133a	2.38 (1.15–7.05)	1.47 (0.97–4.12)	6.31 (1.65–11.91)	1.09 (0.29–2.59)	01907
miR-133b	3.24 (1.92–6.83)	2.26 (1.43–3.19)	4.17 (1.21–8.23)	1.22 (0.65–3.62)	0.2059
miR-206	3.21 (0.96–9.22)	1.02 (0.60–6.29)	4.03 (1.74–17.61)	0.85 (0.36–10.68)	0.4410
miR-208a	1.31 (0.89–3.15)	0.86 (0.67–2.29)	4.84 (0.75–7.10)	0.70 (0.41–4.23)	0.3830
miR-208b	5.37 (1.88–9.09)	4.33 (2.76–6.36)	2.93 (0.77–379.80)	5.32 (1.49–21.16)	0.4765
miR-499	2.22 (1.38–6.55)	1.73 (1.35–3.40)	1.97 (1.51–7.13)	1.62 (0.89–2.54)	0.4023

*The serum miRNA levels in MD-carriers with different gene mutation did not conform to the normal distribution and were expressed by the median and IQR. The non-parametric Mann–Whitney U-test was used for comparison between the two groups. P < 0.05 was considered statistically significant*.

### The Correlations Between Serum Levels of MyomiRs, Age and CK Levels

In the MD-carrier group, no significant correlation between serum miRNAs and age was found. When we assessed the relationship between serum miRNAs and CK in the MD-carrier group, no significant relationship was found but miR-1 (Spearman's rho = +0.406 *p* = 0.017) showed a weak but significant correlation (Table 4).

**Table 4:** Correlation between serum miRNA levels and age and CK levels of MD-carriers.

**MD-carriers (*****n*** **=** **34)**		**Age, years**	**CK, U/L**
miR-1	*r*-value	0.158	0.406^*^
	*p*-value	0.371	**0.017**
miR-133a	*r*-value	−0.037	0.020
	*p*-value	0.836	0.912
miR-133b	*r*-value	−0.013	0.130
	*p*-value	0.940	0.463
miR-206	*r*-value	−0.047	−0.157
	*p*-value	0.790	0.375
miR-208a	*r*-value	0.024	0.024
	*p*-value	0.894	0.895
miR-208b	*r*-value	0.033	0.042
	*p*-value	0.853	0.815
miR-499	*r*-value	−0.051	0.142
	*p*-value	0.774	0.422

* *weak correlation. r-value: correlation coefficient*.

*The non-parametric Spearman correlation coefficient was used to analyze the correlation between the serum miRNA levels of MD-carriers and age and CK levels. P-value in bold was statistically significant (p < 0.05)*.

### Assessment of the Diagnostic Potential of MyomiRs in MD-Carriers

**Figure 2:**
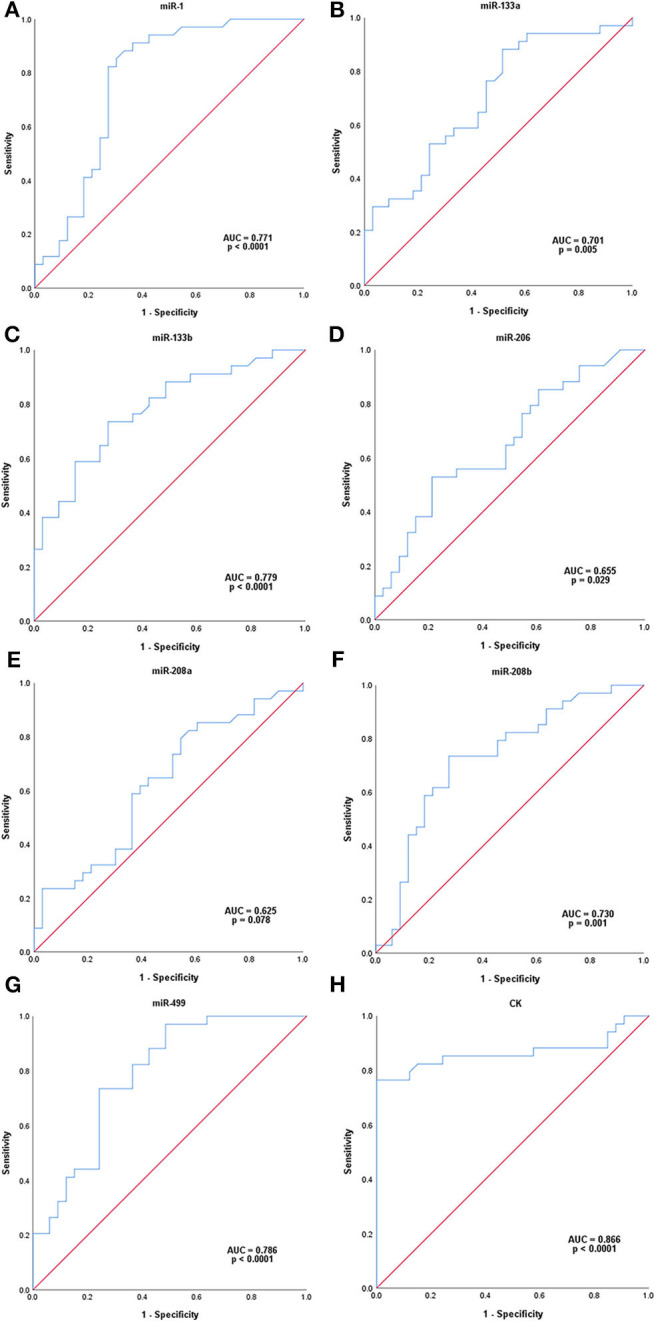
ROC analysis of serum microRNAs levels in MD-carriers. **(A-H)**; miR-1, miR-133a, miR-133b, miR-206, miR-208a, miR-208b, mir-499, and CK, respectively. *P*-value indicated asymptotic significance, and *p* < 0.05 was considered statistically significant.

ROC analysis was carried out to assess the capacity of serum miRNAs to identify female MD-carriers and controls (Figure 2). Our data indicated that five of the seven up-regulated miRNAs in MD-carriers vs. controls revealed AUC values exceeding 70.0%, and the other two exceeding 60.0%. AUC, sensitivity and specificity for these miRNAs were, respectively, listed as follows: (a) miR-499: 78.6, 73.5, and 75.8% (with a cut-off value of 1.485, *p* < 0.0001), (b) miR-133b: 77.9, 73.5, and 72.7% (with a cut-off value of 1.760, *p* < 0.0001), (c) miR-1: 77.1, 82.4, and 72.7% (with a cut-off value of 1.215, *p* < 0.0001), (d) miR-208b: 73.0, 73.5, and 72.7 (with a cut-off value of 2.555, *p* = 0.001), (e) miR-133a: 70.1, 88.2, and 48.5% (with a cut-off value of 0.690, *p* = 0.005), (f) miR-206: 65.5, 52.9, and 78.8 (with a cut-off value of 2.645, *p* = 0.029) and (g) miR-208a: 62.5, 79.4, and 45.5% (with a cut-off value of 0.700, *p* = 0.078). In comparison, ROC analysis for the conventional serum marker, CK, with regards to the identification of MD-carriers revealed an AUC value of 86.6% with a sensitivity of 76.5%, a specificity of 100.0% (with a cut-off value of 146.500, *p* < 0.0001) (Figure 2). None of the seven miRNAs had a higher AUC and specificity than CK, but the sensitivity of miR-1 and miR-133a was higher than CK.

### Serum Levels of MyomiRs in MD-Carriers With Normal and Elevated CK Levels

**Figure 3:**
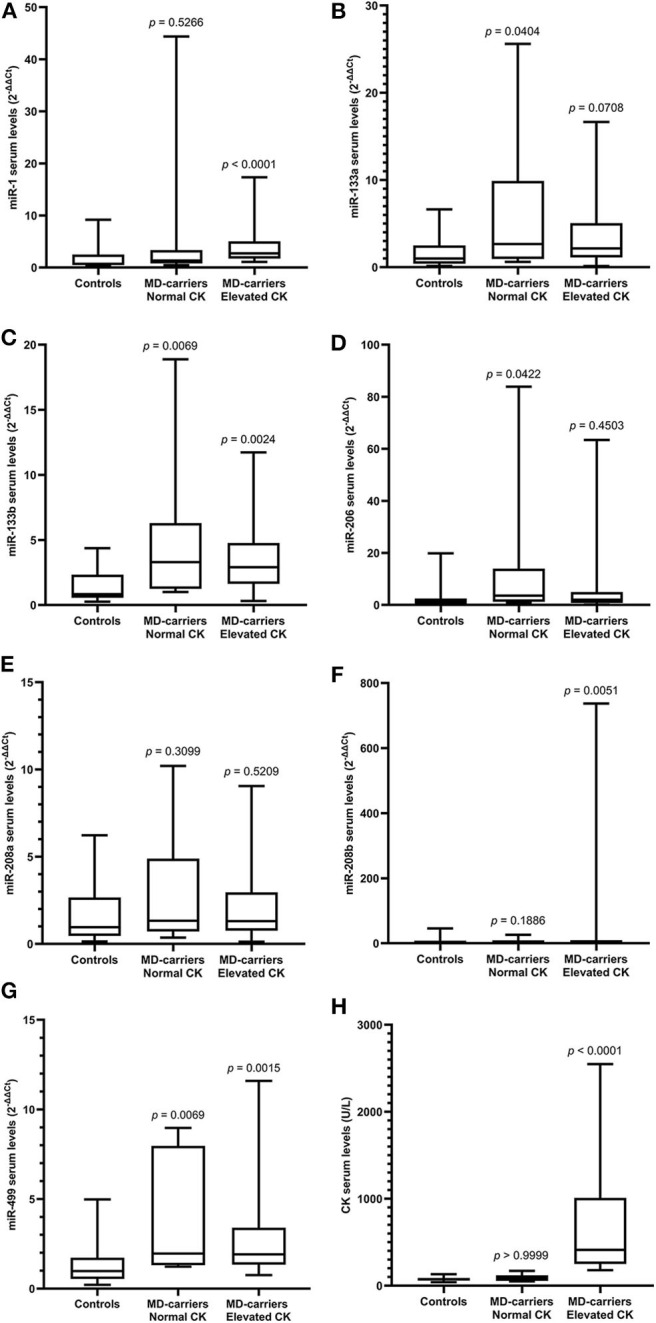
Expression of circulating miR-1 **(A)**, miR-133a **(B)**, miR-133b **(C)**, miR-206 **(D)**, miR-208a **(E)**, miR-208b **(F)**, mir-499 **(G)**, and CK **(H)** levels in serum of MD-carriers with normal CK values (*n* = 10) and MD-carriers with elevated CK values (*n* = 24) as well as control subjects (*n* = 33). As data of the above three groups were non-normally distributed variables, the non-parametric Kruskal–Wallis *H*-test was used for multiple comparison. *P*-value was the comparison of serum miRNA levels in MD-carriers with normal CK or elevated CK vs. controls. *P* < 0.05 was considered statistically significant.

Serum miRNAs in MD-carriers with normal and elevated CK levels are shown in Table 5. A varying degree of up-regulation was found in MD-carriers with normal CK (*n* = 10) compared to controls (*n* = 33) for all serum miRNAs included in the analysis (Figure 3): miR-1 (1.89-fold increase, *p* = 0.5266), miR-133a (2.70-fold, *p* = 0.0404), miR-133b (3.99-fold, *p* = 0.0069), miR-206 (3.02-fold, *p* = 0.0422), miR-208a (1.40-fold, *p* = 0.3099), miR-208b (2.84-fold, *p* = 0.1866) and miR-499 (2.00-fold, *p* = 0.0069). Similarly, compared with the control group, the serum miRNAs in MD-carriers with elevated CK levels (*n* = 24) were also increased to varying degrees (Figure 3): miR-1 (3.82-fold increase, *p* < 0.0001), miR-133a (2.18-fold, *p* = 0.0708), miR-133b (3.51-fold, *p* = 0.0024), miR-206 (1.70-fold, *p* = 0.4503), miR-208a (1.36-fold, *p* =0.5209), miR-208b (3.87-fold, *p* = 0.0051) and miR-499 (1.96-fold, *p* = 0.0015). Among them, a significant up-regulation was found for miR-133b and miR-499, both in CK elevated MD-carriers and in those patients with normal CK levels.

**Table 5:** The characteristics of MD-carriers and serum miRNA levels in relation to CK values.

	**Normal CK**	**Elevated CK**	***p*-value**
Female, *n*	10	24	–
DMD/BMD, *n* (%)	9 (90.00)/1 (10.00)	24 (100.00)/0 (0.00)	–
Age, years (mean ± SD)	31.00 ± 6.65	31.25 ± 9.79	0.942
CK, U/L [median (IQR)]	77.00 (55.25–120.50)	412.50 (250.50–1011.00)	**<** **0.0001**
miR-1 (2^−^^ΔΔCt^) [median (IQR)]	1.34 (0.79–3.36)	2.71 (1.79–5.05)	0.2835
miR-133a (2^−^^ΔΔCt^) [median (IQR)]	2.67 (0.94–9.89)	2.16 (1.13–5.06)	> 0.9999
miR-133b (2^−^^ΔΔCt^) [median (IQR)]	3.31 (1.25–6.30)	2.91 (1.63–4.78)	> 0.9999
miR-206 (2^−^^ΔΔCt^) [median (IQR)]	3.56 (1.22–13.94)	2.01 (0.74–4.95)	0.5511
miR-208a (2^−^^ΔΔCt^) [median (IQR)]	1.34 (0.72–4.90)	1.31 (0.76–2.96)	> 0.9999
miR-208b (2^−^^ΔΔCt^) [median (IQR)]	3.58 (1.04–8.94)	4.87 (2.03–8.88)	> 0.9999
miR-499 (2^−^^ΔΔCt^) [median (IQR)]	1.96 (1.31–7.97)	1.92 (1.38–3.41)	> 0.9999

*The number of females in the two groups was expressed by frequency (n). The classification of DMD/BMD was expressed in frequency (n) and percentage (%). The age of the MD-carriers conformed to a normal distribution and was expressed as mean ± SD, and the independent-sample t-test was used for analysis. CK and serum miRNA levels did not conform to the normal distribution and was expressed in median and IQR, and the non-parametric Mann-Whitney U-test was used for analysis. P-value in bold are statistically significant (p < 0.05)*.

### Assessment of the Diagnostic Potential of the Combination of MyomiRs and CK in MD-Carriers

**Figure 4:**
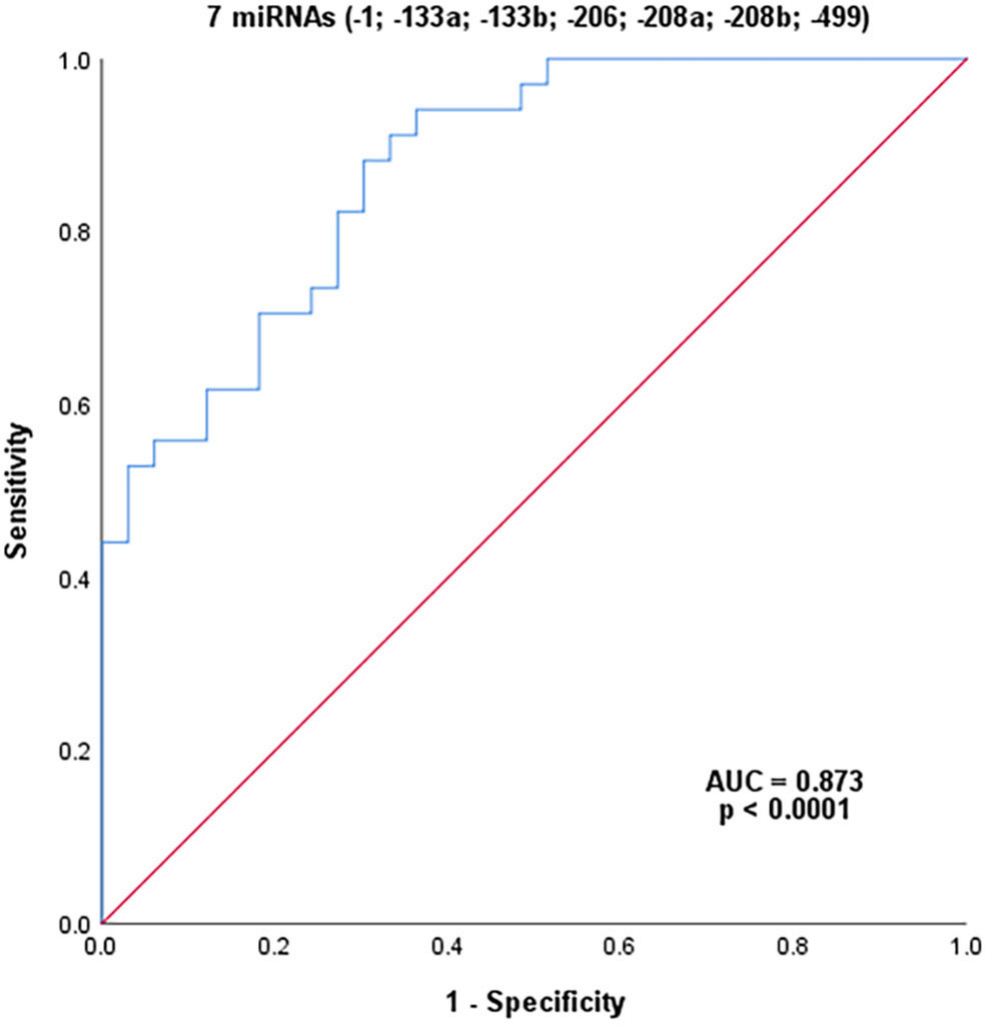
ROC analysis of the combination of seven serum miRNAs. *P*-value indicated asymptotic significance, and *p* < 0.05 was considered statistically significant.

Combining all seven myomiRs (miR-1, miR-133a, miR-133b, miR-206, miR-208a, miR-208b, and miR-499; Figure 4) as potential diagnostic signatures for female MD-carriers, an improved AUC value of 87.3% was reached with a sensitivity of 91.2% and a specificity of 66.7% (with a cut-off value of 0.339, *p* < 0.0001). In addition to specificity, this combination had higher sensitivity and AUC value than CK alone or any single miRNA.

**Figure 5:**
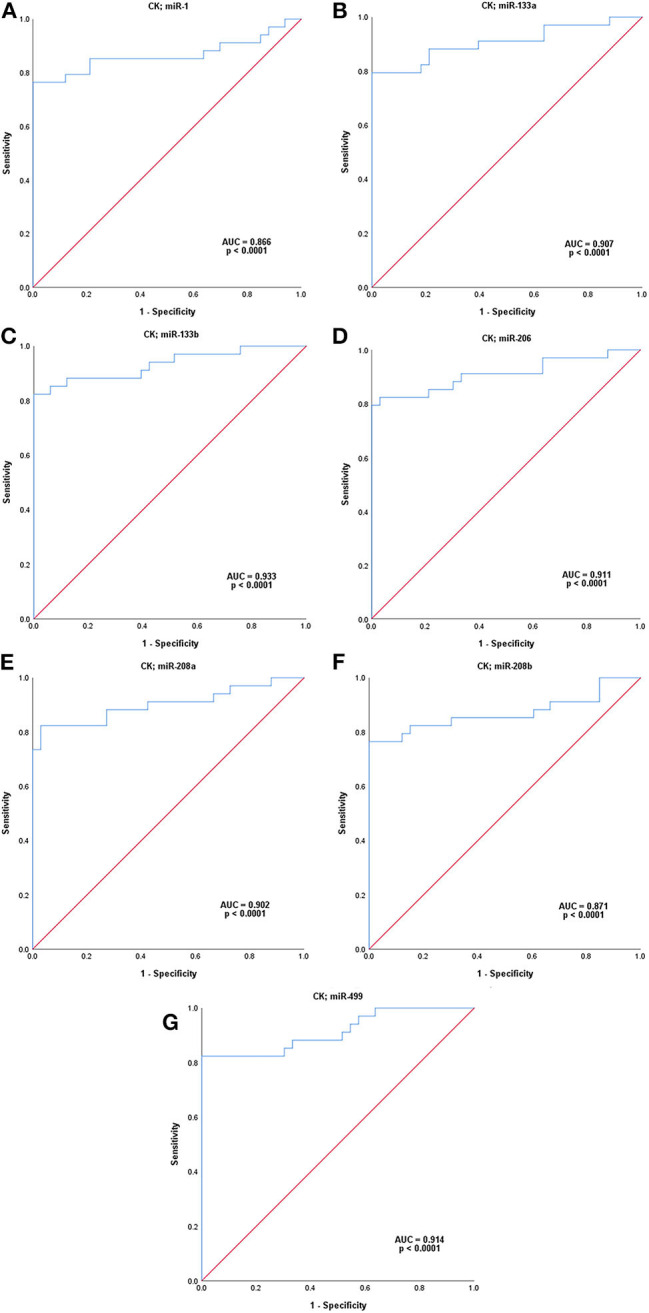
ROC analysis of seven serum microRNA levels combined with those of CK, respectively. **(A–G)**; miR-1, miR-133a, miR-133b, miR-206, miR-208a, miR-208b, and miR-499. *P*-value indicated asymptotic significance, and *p* < 0.05 was considered statistically significant.

In order to further explore the potential of myomiRs to diagnose MD-carriers, we performed ROC analysis by combining CK with the seven different myomiRs, respectively (Figure 5). Their AUCs, sensitivities and specificities were all improved compared to each individual evaluation. Among which, the AUC values of the combination of CK with miR-133b (AUC 93.3%, sensitivity 82.4%, specificity 100.0%, with a cut-off value of 0.618) and CK with miR-499 (AUC 91.4%, sensitivity 82.4%, specificity 100.0%, with a cut-off value of 0.594) exceeded 90.0%, the sensitivities exceeded 80.0% and the specificities were 100.0%. The combination of these two miRNAs with CK had an even higher AUC value and sensitivity than CK or any individual miRNA, and the specificity was also 100%.

### Assessment of Possible Predictors of MD-Carriers

In order to identify potential predictors for the occurrence of MD-carriers in the population, we first performed binary logistic regression analyses for a series of parameters, including CK and the seven miRNAs (Table 6). In this analysis, a significant association with the presence of MD-carriers was found for the CK, miR-1, miR-133a, miR-133b, and miR-499. Subsequently, we performed a multivariable regression analysis focusing on CK and these four miRNAs. In this model, CK (OR 0.406, 95% CI 0.000–0.001, *p* < 0.0001) and miR-499 (OR 0.323, 95% CI 0.023–0.106, *p* = 0.003) were considered to be independent predictors for the presence of MD-carriers.

**Table 6:** Binary logistic regression analysis regarding the predictors for MD-carriers.

**Variable (*n* = 67)**	**OR (95 % CI)**	***p*-value**
CK	1.028 (1.011–1.045)	**0.001**
miR-1	1.254 (1.003–1.568)	**0.047**
miR-133a	1.312 (1.034–1.666)	**0.025**
miR-133b	1.939 (1.286–2.924)	**0.002**
miR-206	1.077 (0.976–1.187)	0.139
miR-208a	1.233 (0.953–1.596)	0.112
miR-208b	1.034 (0.965–1.107)	0.341
miR-499	1.814 (1.172–2.809)	**0.008**

*OR, odds ratio = Exp(Beta); CI, confidence interval*.

*P-value in bold was statistically significant (p < 0.05). The OR values of CK, miR-1, miR-133a, miR-133b, and miR-499 was >1 and their p < 0.05*.

## Discussion

DMD is an X-linked recessive hereditary muscle disease. Detection of female carriers is important to avoid the birth of affected children (4). CK is currently the most common used non-invasive biomarker in detecting carriers, but since CK is not always reliable and only 45.0–76.0% of female carriers have increased levels of CK, the search for new biomarkers in detecting DMD/BMD carriers is especially important (5–7, 22). At present, there are also some studies on miRNAs as biomarkers of DMD, but few are about DMD carriers. In the present study, we tested the serum expression levels of seven miRNAs to evaluate their potential for diagnosing DMD/BMD female carriers.

The results of this study showed that these seven myomiRs were highly expressed (about 2.0–3.5 fold) in the serum of MD-carriers, in comparison to the control group. Cacchiarelli et al. (27) found that miR-1, miR-133, and miR-206 expression were 5–100 fold enriched in DMD and BMD patients in comparison to healthy controls. They concluded that the differences in serum levels of these miRNAs were due to the intensive degeneration that occurred in the muscles of MD patients. Hu et al. (13) considered that the increase of muscle-specific miRNAs in serum was explained by the observed miRNAs in exosomal particles (28), which were in turn caused by an increased leakage or secretion of miRNAs from muscles (29). Israeli et al. (10) considered that the upregulation of miR-1, miR-133, and miR-206 in the serum of MD patients was due to their high expression in muscles and the upregulation was proportional to the levels of myofiber degeneration. Therefore, we speculated that damage of the muscles of MD-carriers in our study led to the release of myomiRs into the circulation. Papa et al. (30) stated that although usually asymptomatic, female carriers of DMD might develop muscle weakness in up to 17.0% of cases, these patients consequently showed signs of a degenerative process upon histological muscle examination regardless of their clinical phenotypes. Preusse et al. (8) also found that muscle biopsies from female carriers of DMD showed morphological abnormalities and a mosaic pattern of dystrophin loss. When studying plasma miRNAs expression in DMD patients and carriers, Mousa et al. (23) speculated that partial muscle functions of the carriers were impaired due to the presence of mutated copies of the DMD gene, and progressive death of muscle cells or modulation of their expression by myocytes could cause variations in the circulating levels of these miRNAs. Based on the above literature reports, we drew the conclusion that although female carriers in the present study had no clinical manifestations of MD, their muscles might have been damaged due to presence of mutated copies of the DMD gene. Subsequently, these myomiRs were released into the circulation, resulting in the higher expression of the seven miRNAs in the serum of female carriers in comparison to the controls group.

We performed a correlation analysis and found that miR-1 and CK had a weak positive correlation in the MD-carriers group. CK is a dimeric enzyme presented within the muscle cytoplasm. CK locates on myofibrils and inside mitochondria (31), and it leaks into the blood stream upon muscle damage (10). Mizuno et al. (29) found that the serum levels of miR-1, miR-133a, and miR-206 were increased in both dystrophin-deficient MD mouse model (mdx) and the canine X-linked MD Japanese dog model (CXMD_J_). However, miR-1, miR-133a, and miR-206 were significantly decreased in the skeletal muscles of mdx mice. They concluded that the increase of muscle-specific miRNAs in the serum of these DMD models was caused by an increase in leakage or secretion of miRNAs from the muscles (29). In this way, the release mechanism of miRNAs and CK seems to be very similar, which may be the reason why miR-1 and CK are weakly and positively correlated in the MD-carriers group. In addition, miR-1 can be expressed in both cardiac and skeletal muscles, and almost exclusively found in mature myofibers (10, 19, 27), which may be another reason for this result.

Recent studies have shown that circulating miRNAs can be used as potential diagnostic biomarkers for female DMD/BMD carriers (4, 11). Therefore, we evaluated the ability of these seven serum myomiRs and CK to diagnose MD-carriers using ROC curve analysis. The results showed that the AUC of CK was the highest, reaching 86.6%, and the AUCs of miR-499, miR-133b, miR-1, miR-208b, and miR-133a were 78.6, 77.9, 77.1, 73.0, and 70.1%, respectively, all of which exceeded 70.0%. In addition, the specificity of CK was 100.0%, which was not exceeded by any miRNA. The sensitivities of miR-133a (88.2%), miR-1 (82.4%), and miR-208a (79.4%) were higher than that of CK, and miR-499 (73.5%) and miR-133b (73.5%) were close to CK. The sensitivity of CK was only 76.5%, which indicated that the false negative rate of CK was higher and it was, therefore, more prone to misdiagnosis. Hashim et al. (32) found that the sensitivity of CK was only 33.3% and the specificity was 100.0% in the diagnosis of female carriers of DMD. To reduce missed diagnosis, we anticipate that new biomarkers may ideally have higher sensitivities than CK. Considering the AUCs, sensitivities and specificities, we suggest that miR-499 (AUC = 78.6%, sensitivity = 73.5%, specificity = 75.8%), miR-133b (AUC = 77.9%, sensitivity = 73.5%, specificity = 72.7%) and miR-1 (AUC = 77.1%, sensitivity = 82.4%, specificity = 72.7%) are potentially new biomarkers which are closest or superior to CK (AUC = 86.6%, sensitivity = 76.5%, specificity = 100.0%).

Anaya-Segura et al. (4) conducted a similar study exploring the diagnostic ability of miR-206 on 23 DMD carriers and 24 controls, and found an AUC of 80.3%, a sensitivity of 78.26% and a specificity of 70.83%. They believed that miR-206 was superior to CK and could be used to detect female carriers of DMD. However, in the present study, the AUC value of miR-206 was only 69.8%, the sensitivity was 48.4% and the specificity was 90.0 %. In addition, Mousa et al. (23) found that miR-499 could be detected in circulating plasma of DMD carriers, while it was undetectable in the controls with a study of 26 DMD carriers and 10 normal controls. ROC curve analysis showed that the specificity and sensitivity of miR-499 were 100.0%. Finally, they concluded that miR-499 could identify the female carriers. This is very similar to our result. Still, experiments with more carriers enrolled will be needed to identify the most appropriate biomarker. Subsequently, we measured serum levels of myomiRs in MD-carriers with normal or elevated CK levels. The results showed that compared with the control group, these seven miRNAs increased in varying degrees in MD-carriers with both normal or elevated levels of CK. Among them, miR-133b and miR-499 in both groups were more significantly increased (*p* < 0.05). This result further suggests that serum myomiRs have the potential to diagnose MD-carriers with normal CK.

Since all seven miRNAs increased to varying degrees in the MD-carriers, ROC curve analysis was performed to evaluate the diagnostic ability of these miRNAs when they were combined. Their AUC was 87.3%, the sensitivity was 91.2% and the specificity was 66.7%. Although the specificity was still lower than CK alone, both the AUC and sensitivity were higher than CK alone or with any single miRNA. In addition, we also combined each miRNA with CK for the ROC analysis. Their AUCs, sensitivities and specificities were improved compared to the individual miRNA or CK. Among them, the AUCs of the combination of CK and miR-133b or miR-499 both exceeded 90.0%, the sensitivities exceeded 80.0% and the specificities were 100.0%. This result suggests that we may be able to use a combination of CK with various miRNAs to diagnose MD-carriers in the future.

Additional binary logistic regression analysis revealed a significant association between the expression levels of CK, miR-1, miR-133a, miR-133b, and miR-499 to the status of MD-carriers. This result is very similar to the miRNAs we screened for by ROC curve analysis. Furthermore, multivariable regression analysis revealed that CK and miR-499 were considered to be independent predictors for MD-carrier status. This result further affirmed the diagnostic ability of miR-499. Combining all of the above data, we suggest that the up-regulation of miR-133b and miR-499 expression in the serum may serve as potential useful biomarkers for identification of MD-carriers (including those with normal CK levels), with miR-499 being the strongest and significant candidate biomarker.

## Conclusion

By detecting each serum level of miR-1, miR-133a, miR-133b, miR-206, miR-208a, miR-208b, and miR-499, we found that these seven myomiRs were up-regulated in MD-carriers, respectively. Serum miR-133b and miR-499 appear to be the promising novel biomarkers for MD-carriers (including those MD-carriers with normal CK levels). In addition, the combination of these seven serum miRNAs and their respective combinations with CK have better diagnostic ability for MD-carriers than CK alone or individual miRNA. Our data suggest that a combination of miRNAs or a combination of CK and miRNAs may be more effective and reliable than any single miRNA and can be used as new non-invasive biomarkers for detecting MD-carriers.

## Data Availability Statement

The raw data supporting the conclusions of this article will be made available by the authors, without undue reservation.

## Ethics Statement

The studies involving human participants were reviewed and approved by Ethics Committee of the First Affiliated Hospital of Guangxi Medical University. Written informed consent to participate in this study was provided by the participants' legal guardian/next of kin.

## Author Contributions

DL and JZha summarized all the information and analyzed the results and together with JZho drafted the manuscript. JZha and QM performed the experiments. MZ, XQ, XN, JM, YH, and DZ collected the specimens and clinical information. All the authors read and approved the final manuscript.

## Conflict of Interest

The authors declare that the research was conducted in the absence of any commercial or financial relationships that could be construed as a potential conflict of interest.

## Abbreviations

AUC: Areas under the curve
BMD: Becker Muscular Dystrophy
CK: Creatine kinase
CMR: Cardiac magnetic resonance imaging
CI: Confidence interval
CXMD_J_: canine X-linked MD Japanese dog model
DAPC: Dystrophin-associated protein complex
DMD: Duchenne Muscular Dystrophy
FPR: False-positive rate
IQR: Interquartile range
MD: Muscular dystrophy
Mdx: MD mouse model
MiRNAs: MicroRNAs
MLPA: Multiplex ligation-dependent probe amplification
MRC: Medical Research Council
MRI: Magnetic resonance imaging
NGS: Next-generation sequencing
OR: Odds ratio
qRT-PCR: Quantitative reverse transcription polymerase chain reaction
ROC: Receiver operating characteristic curve
SD: Standard deviation.

## References

[1] Emery AE. Population frequencies of inherited neuromuscular diseases–a world survey. Neuromuscul Disord. (1991) 1:19–29. 10.1016/0960-8966(91)90039-U 1822774

[2] Mercier S Toutain A Toussaint A Raynaud M de Barace C Marcorelles P. Genetic and clinical specificity of 26 symptomatic carriers for dystrophinopathies at pediatric age. Eur J Hum Genet. (2013) 21:855–63. 10.1038/ejhg.2012.269 23299919 PMC3722679

[3] Imbornoni L Price ET Andrews J Meaney FJ Ciafaloni E Cunniff C Diagnostic and clinical characteristics of early-manifesting females with duchenne or becker muscular dystrophy. Am J Med Genet A. (2014) 164a:2769–74. 10.1002/ajmg.a.36728 25125379

[4] Anaya-Segura MA Rangel-Villalobos H Martinez-Cortes G Gomez-Diaz B Coral-Vazquez R M Zamora-Gonzalez EO. Serum levels of microRNA-206 and novel mini-STR assays for carrier detection in duchenne muscular dystrophy. Int J Mol Sci. (2016) 17:1334. 10.3390/ijms17081334 27529242 PMC5000731

[5] Wang Q Yang X Yan Y Song N Lin C Jin C Duchenne or becker muscular dystrophy: a clinical, genetic and immunohistochemical study in China. Neurol India. (2011) 59:797–802. 10.4103/0028-3886.91354 22234188

[6] Zhong J Xie Y Bhandari V Chen G Dang Y Liao H. Clinical and genetic characteristics of female dystrophinopathy carriers. Mol Med Rep. (2019) 19:3035–44. 10.3892/mmr.2019.9982 30816495 PMC6423608

[7] Hoogerwaard E M Bakker E Ippel P F Oosterwijk J C Majoor-Krakauer D F Leschot N J. Signs and symptoms of duchenne muscular dystrophy and becker muscular dystrophy among carriers in the Netherlands: a cohort study. Lancet. (1999) 353:2116–9. 10.1016/S0140-6736(98)10028-4 10382696

[8] Preusse C Von Moers A Kolbel H Pehl D Goebel H H Schara U. Inflammation-induced fibrosis in skeletal muscle of female carriers of duchenne muscular dystrophy. Neuromuscul Disord. (2019) 29:487–96. 10.1016/j.nmd.2019.05.003 31326192

[9] Gasper MC Gilchrist JM. Creatine kinase: a review of its use in the diagnosis of muscle disease. Med Health R I. (2005) 88:400–394. 16363394

[10] Israeli D Poupiot J Amor F Charton K Lostal W Jeanson-Leh L. Circulating miRNAs are generic and versatile therapeutic monitoring biomarkers in muscular dystrophies. Sci Rep. (2016) 6:28097. 10.1038/srep28097 27323895 PMC4914855

[11] Florian A Patrascu A Tremmel R Rosch S Sechtem U Schwab M. Identification of cardiomyopathy-associated circulating mirna biomarkers in muscular dystrophy female carriers using a complementary cardiac imaging and plasma profiling approach. Front Physiol. (2018) 9:1770. 10.3389/fphys.2018.01770 30622476 PMC6308188

[12] Chen K Rajewsky N The evolution of gene regulation by transcription factors and microRNAs. Nat Rev Genet. (2007) 8:93–103. 10.1038/nrg1990 17230196

[13] Hu J Kong M Ye Y Hong S Cheng L Jiang L. Serum miR-206 and other muscle-specific microRNAs as non-invasive biomarkers for duchenne muscular dystrophy. J Neurochem. (2014) 129:877–83. 10.1111/jnc.12662 24460924

[14] Chen X Ba Y Ma L Cai X Yin Y Wang K. Characterization of microRNAs in serum: a novel class of biomarkers for diagnosis of cancer and other diseases. Cell Res. (2008) 18:997–1006. 10.1038/cr.2008.282 18766170

[15] Mccarthy JJ. MicroRNA-206: the skeletal muscle-specific myomiR. Biochim Biophys Acta. (2008) 1779:682–91. 10.1016/j.bbagrm.2008.03.001 18381085 PMC2656394

[16] Sempere L F Freemantle S Pitha-Rowe I Moss E Dmitrovsky E Ambros V. Expression profiling of mammalian microRNAs uncovers a subset of brain-expressed microRNAs with possible roles in murine and human neuronal differentiation. Genome Biol. (2004) 5:R13. 10.1186/gb-2004-5-3-r13 15003116 PMC395763

[17] Van Rooij E Sutherland LB Qi X Richardson J A Hill J Olson EN. Control of stress-dependent cardiac growth and gene expression by a microRNA. Science. (2007) 316:575–579. 10.1126/science.1139089 17379774

[18] Van Rooij E Quiat D Johnson B A Sutherland L B Qi X Richardson J A. A family of microRNAs encoded by myosin genes governs myosin expression and muscle performance. Dev Cell. (2009) 17:662–73. 10.1016/j.devcel.2009.10.013 19922871 PMC2796371

[19] Horak M Novak J Bienertova-Vasku J. Muscle-specific microRNAs in skeletal muscle development. Dev Biol. (2016) 410:1–13. 10.1016/j.ydbio.2015.12.013 26708096

[20] Small EM O'rourke JR Moresi V Sutherland LB Mcanally J Gerard RD. Regulation of PI3-kinase/Akt signaling by muscle-enriched microRNA-486. Proc Natl Acad Sci USA. (2010). 107:4218–23. 10.1073/pnas.1000300107 20142475 PMC2840099

[21] Zilahi E Adamecz Z Bodoki L Griger Z Poliska S Nagy-Vincze M. Dysregulated expression profile of myomiRs in the skeletal muscle of patients with polymyositis. Ejifcc. (2019) 30:237–45. 31372109 PMC6599196

[22] Li X Li Y Zhao L Zhang D Yao X Zhang H. Circulating muscle-specific miRNAs in duchenne muscular dystrophy patients. Mol Ther Nucleic Acids. (2014) 3:e177. 10.1038/mtna.2014.29 25050825 PMC4121518

[23] Mousa N O Abdellatif A Fahmy N Zada S El-Fawal H Osman A. Circulating microRNAs in duchenne muscular dystrophy. Clin Neurol Neurosurg. (2020) 189:105634. 10.1016/j.clineuro.2019.105634 31838454

[24] Scott O M Hyde S A Goddard C Dubowitz V. Quantitation of muscle function in children: a prospective study in duchenne muscular dystrophy. Muscle Nerve. (1982) 5:291–301. 10.1002/mus.880050405 7099196

[25] Obuchowski NA ROC analysis. AJR Am J Roentgenol. (2005). 184:364–72. 10.2214/ajr.184.2.01840364 15671347

[26] Søreide K. Receiver-operating characteristic curve analysis in diagnostic, prognostic and predictive biomarker research. J Clin Pathol. (2009) 62:1–5. 10.1136/jcp.2008.061010 18818262

[27] Cacchiarelli D Legnini I Martone J Cazzella V D'amico A Bertini E. miRNAs as serum biomarkers for duchenne muscular dystrophy. EMBO Mol Med. (2011) 3:258–65. 10.1002/emmm.201100133 21425469 PMC3112257

[28] Mitchell PS Parkin RK Kroh EM Fritz BR Wyman SK Pogosova-Agadjanyan EL. Circulating microRNAs as stable blood-based markers for cancer detection. Proc Natl Acad Sci USA. (2008) 105:10513–18. 10.1073/pnas.0804549105 18663219 PMC2492472

[29] Mizuno H Nakamura A Aoki Y Ito N Kishi S Yamamoto K. Identification of muscle-specific microRNAs in serum of muscular dystrophy animal models: promising novel blood-based markers for muscular dystrophy. PLoS ONE. (2011) 6:e18388. 10.1371/journal.pone.0018388 21479190 PMC3068182

[30] Papa R Madia F Bartolomeo D Trucco F Pedemonte M Traverso M. Genetic and early clinical manifestations of females heterozygous for duchenne/becker muscular dystrophy. Pediatr Neurol. (2016) 55:58–63. 10.1016/j.pediatrneurol.2015.11.004 26718981

[31] Chakrabarty T Tirupathi S Thompson A. How to use: creatine kinase. Arch Dis Child Educ Pract Ed. (2019) 105:157–63. 10.1136/archdischild-2019-317184 31296557

[32] Hashim R Shaheen S Ahmad S Sattar A Khan FA. Comparison of serum creatine kinase estimation with short tandem repeats based linkage analysis in carriers and affected children of Duchenne muscular dystrophy. J Ayub Med Coll Abbottabad. (2011) 23:125–8. 22830166

