# Corrigendum: Serum MyomiRs as Biomarkers for Female Carriers of Duchenne/Becker Muscular Dystrophy

**DOI:** 10.3389/fneur.2020.617878

**Published:** 2020-11-12

**Authors:** Jiapeng Zhang, Qi Meng, Jingzi Zhong, Min Zhang, Xiao Qin, Xiaohua Ni, Jiawen Ma, Yangwen He, Dan Zeng, Dan Lan

**Affiliations:** Department of Pediatrics, The First Affiliated Hospital of Guangxi Medical University, Nanning, China

**Keywords:** duchenne/becker muscular dystrophy, female carriers, microRNAs, myomiRs, creatine kinase, biomarkers

In the original article, there was an error. The cut-off value of the ROC curve was wrong, and it was accidentally written as the value of the Youden's index.

A correction has been made to *Results, Assessment of the Diagnostic Potential of MyomiRs in MD-Carriers, Paragraph 1*. The corrected paragraph is written below:

ROC analysis was carried out to assess the capacity of serum miRNAs to identify female MD-carriers and controls (**Figure 2**). Our data indicated that five of the seven up-regulated miRNAs in MD-carriers vs. controls revealed AUC values exceeding 70.0%, and the other two exceeding 60.0%. AUC, sensitivity and specificity for these miRNAs were, respectively, listed as follows: (a) miR-499: 78.6, 73.5, and 75.8% (with a cut-off value of 1.485, *p* < 0.0001), (b) miR-133b: 77.9, 73.5, and 72.7% (with a cut-off value of 1.760, *p* < 0.0001), (c) miR-1: 77.1, 82.4, and 72.7% (with a cut-off value of 1.215, *p* < 0.0001), (d) miR-208b: 73.0, 73.5, and 72.7 (with a cut-off value of 2.555, *p* = 0.001), (e) miR-133a: 70.1, 88.2, and 48.5% (with a cut-off value of 0.690, *p* = 0.005), (f) miR-206: 65.5, 52.9, and 78.8 (with a cut-off value of 2.645, *p* = 0.029) and (g) miR-208a: 62.5, 79.4, and 45.5% (with a cut-off value of 0.700, *p* = 0.078). In comparison, ROC analysis for the conventional serum marker, CK, with regards to the identification of MD-carriers revealed an AUC value of 86.6% with a sensitivity of 76.5%, a specificity of 100.0% (with a cut-off value of 146.500, *p* < 0.0001) (**Figure 2**). None of the seven miRNAs had a higher AUC and specificity than CK, but the sensitivity of miR-1 and miR-133a was higher than CK.

Corrections have also been made to *Results, Assessment of the Diagnostic Potential of the Combination of MyomiRs and CK in MD-Carriers, Paragraphs 1 and 2*. The corrected paragraphs are shown below:

Combining all seven myomiRs (miR-1, miR-133a, miR-133b, miR-206, miR-208a, miR-208b, and miR-499; **Figure 4**) as potential diagnostic signatures for female MD-carriers, an improved AUC value of 87.3% was reached with a sensitivity of 91.2% and a specificity of 66.7% (with a cut-off value of 0.339, *p* < 0.0001). In addition to specificity, this combination had higher sensitivity and AUC value than CK alone or any single miRNA.

In order to further explore the potential of myomiRs to diagnose MD-carriers, we performed ROC analysis by combining CK with the seven different myomiRs, respectively (**Figure 5**). Their AUCs, sensitivities and specificities were all improved compared to each individual evaluation. Among which, the AUC values of the combination of CK with miR-133b (AUC 93.3%, sensitivity 82.4%, specificity 100.0%, with a cut-off value of 0.618) and CK with miR-499 (AUC 91.4%, sensitivity 82.4%, specificity 100.0%, with a cut-off value of 0.594) exceeded 90.0%, the sensitivities exceeded 80.0% and the specificities were 100.0%. The combination of these two miRNAs with CK had an even higher AUC value and sensitivity than CK or any individual miRNA, and the specificity was also 100%.

The authors apologize for these errors and state that they do not change the scientific conclusions of the article in any way. The original article has been updated.

