# Supplementary material for: Serum MyomiRs as Biomarkers for Female Carriers of Duchenne/Becker Muscular Dystrophy
Source: Front Neurol. 2020 Sep 18;11:563609. doi: 10.3389/fneur.2020.563609 (PMC7530632; doi:10.3389/fneur.2020.563609)
Supplement: Supplementary file 1 [file Table_1.docx]

**Supplementary file**. DMD gene mutation sites in all carriers.

| **Deletion mutation** | **Duplication mutation** | **Point mutation** | **Splice mutation** |
| --- | --- | --- | --- |
| del EX49-50 | dup EX2-9 | c.7285delG | c.831+1G>T |
| del EX48-55 | dup EX12-30 | c.7285delG | c.9551+1G>A |
| del EX45-52 | dup EX44 | c.5287C>T | c.9551+1G>A |
| del EX14-43 | dup EX4-7 | c.8835_8838del | c.3420+1G>A |
| del EX14-43 | dup EX60-62 | c.2560C>T |  |
| del EX3-13 |  |  |  |
| del EX50-52 |  |  |  |
| del EX26-44 |  |  |  |
| del EX49-51 |  |  |  |
| del EX48-52 |  |  |  |
| del EX8-20 |  |  |  |
| del EX49-50 |  |  |  |
| del EX45-52 |  |  |  |
| del EX46-49 |  |  |  |
| del EX48-52 |  |  |  |
| del EX45-49 |  |  |  |
| del EX45-48 |  |  |  |
| del EX45-48 |  |  |  |
| del EX48-54 |  |  |  |
| del EX2-50 |  |  |  |

EX: exon, del: deletion, dup: duplication.
